# CPR in correctional facilities: a missed opportunity?

**DOI:** 10.1186/s40352-022-00202-9

**Published:** 2022-12-27

**Authors:** Christopher Scott Sampson, Julie A. W. Stilley, Elizabeth Kendrick, Kayla Riel

**Affiliations:** Department of Emergency Medicine, University of Missouri School of Medicine, One Hospital Drive, Columbia, MO 65212 USA

**Keywords:** CPR, Correctional facilities, Prisoners

## Abstract

In the incarcerated population, the largest ethnic and racial group is Black people. Heart disease is known as the leading causes of death in the United States which can lead to cardiac arrest. Layperson cardiopulmonary resuscitation (CPR) has been shown to provide a benefit and increase likelihood of return of spontaneous circulation (ROSC). Recent research shows that in witnessed out of hospital cardiac arrests, the likelihood of receiving bystander CPR was found to be less among Black or Hispanic people when compared to White persons. One neglected area for layperson CPR training are these correctional facilities. This population is known to have higher rates of diabetes, high blood pressure and coronary artery disease, all of which contribute to an increased risk of acute coronary syndrome.

A search was performed of the NEMSIS database. When comparing witnessed cardiac arrest, incidents without bystander interventions occurred more frequently than expected if the arrest was witnessed by a family member or other lay person. These interventions included bystander CPR or AED placement with or without defibrillation.

The data presented shows that there is an unmet need of additional lay person CPR training in correctional facilities which could be implemented for little cost.

## Introduction

The total number of people incarcerated in federal, state, local and tribal systems approaches 2 million people. Over half reside in state prisons (1 million) and a quarter in local jails (547,000) (Sawyer & Wagner, 2022). Of this population, the largest ethnic and racial group is Black people who comprise 2306 per 100,000 incarcerated persons (U.S. Incarceration rate by race and ethnicity, 2010). Heart disease is known as the leading causes of death in the United States which can be a contributor to cardiac arrest (Deaths and Mortality, 2022). Layperson cardiopulmonary resuscitation (CPR) has been shown to provide a benefit and increase likelihood of return of spontaneous circulation (ROSC). CPR is an emergency procedure consisting often of chest compressions with a goal of supplying the brain with blood flow until more definitive care can be provided. Recent research shows that in witnessed out of hospital cardiac arrests, the likelihood of receiving bystander CPR was found to be less among Black or Hispanic people when compared to White persons (Garcia et al., 2022). One neglected area for layperson CPR training are correctional facilities. In certain cases of cardiac arrest, the use of an automated external defibrillator (AED) can provide a lifesaving therapy. The AED is able to recognize a shockable cardiac rhythm and provide an electrical shock that will convert the heart rhythm from unstable to stable. There are many factors that lead to a delay in obtaining definitive care through an AED or emergency medical services (EMS). These delays include recognition, access to the patient given security issues, and activation of 911 system. Another important factor is the health status of the incarcerated population. Incarcerated individuals are known to be disproportionately in poor health at all phases of the incarceration cycle (Wildeman & Wang, 2017). This population is known to have higher rates of diabetes, high blood pressure and coronary artery disease, all of which contribute to an increased risk of acute coronary syndrome. Based on previous research it is known that with urban environments, there are socioeconomic disparities when it comes to who is taught layperson CPR with age, race, median household income and educational level all being independent factors (Abdulhay et al., 2019).

## Methods

These recognized issues are supported by the National Emergency Medical Services Information System (NEMSIS) data. NEMSIS is a national database that stores EMS data from across the United States and is able to be searched to determine benchmarks or effectiveness of clinical interventions in the prehospital setting. A search was performed of the NEMSIS database (V3 – Current Plus 2 years; search October 12, 2022). Elements included Cardiac Arrest status, witness status, AED use or CPR care provided prior to EMS arrival, and scene locations including the element “prison”. Gender and race were included with the prior elements in a secondary search (October 24, 2022).

## Results

In the prison population studied, when a cardiac arrest was witnessed by a family member or lay person, bystander intervention did not occur. These interventions included bystander CPR or AED placement with or without defibrillation. Given the setting of these cardiac arrests, a lay person would likely be a correctional facility employee. Statistically this event was found to occur more often than expected (Chi-square, *p* < 0.001) (Table 1). While cardiac arrest victim race did not seem to affect frequency, gender of the patient showed that men received more bystander interventions than expected in all interventions (Table 2). However female cardiac arrest victims had less instances of AED placement whether bystander CPR was initiated or not (Chi-square, *p* < 0.001).

**Table 1:** Summary of all witnessed arrest occurring in locations related to prisons

	Witnessed by	Expected	Observed	Chi-Square
No Intervention (*n* = 258, df = 2)	Healthcare Worker	111	47 (↓)	< 0.001
	Family Member	15	51 (↑)	
	Other Lay Person	133	160 (↑)	
CPR Only (*n* = 403, df = 2)	Healthcare Worker	173	127 (↓)	< 0.001
	Family Member	23	45 (↑)	
	Other Lay Person	207	231 (↑)	
AED Only (*n* = 60, df = 2)	Healthcare Worker	26	19 (≈)	0.017
	Family Member	3	0 (≈)	
	Other Lay Person	31	41 (≈)	
CPR + AED (*n* = 1384, df = 2)	Healthcare Worker	593	696 (↑)	< 0.001
	Family Member	79	25 (↓)	
	Other Lay Person	712	663 (↓)

**Table 2:** Summary of interventions on victims of cardiac arrest by gender occurring in locations related to prisons

**Female** (*n* = 234, df 3)	Expected	Observed	Chi-Square
No Intervention	86	84 (≈)	< 0.001
CPR Only	29	54 (↑)	
AED Only	6	11 (↑)	
CPR + AED	113	85 (↓)	
**Male** (*n* = 1489, df 3)	Expected	Observed	Chi-Square
No Intervention	546	363 (↓)	< 0.001
CPR Only	186	264 (↑)	
AED Only	39	60 (↑)	
CPR + AED	719	810 (↑)	

## Conclusion

The data presented shows that there is an unmet need of additional lay person CPR training in correctional facilities. This training should not only include those who work in the facilities but also those incarcerated, as they are more likely to be the first to encounter a person in cardiac arrest. Training time is minimal with basic CPR certification only taking a few hours to complete and a certification lasting approximately 2 years. Average cost of adult CPR training is around $50 per individual. One study looking at training requirements for correctional officers found that CPR training was only provided in three states (Alaska, Colorado, Oregon) (Kowalski, 2020). This woefully small number illustrates a huge gap in prisoner healthcare. Cardiac arrest bystander disparities known to occur outside the prison system seem to occur inside the prison system as well, though focusing in the limitation of AED use in female prisoners leads to further questions about emergency equipment locations and availability in female prisons. Given an additional rise is substance abuse in correctional facilities, recognition of cardiac arrest or an overdosed state is paramount. Their prompt recognition and initial action, can potentially affect the outcome and alter the course of a life. The implementation of a broad layperson CPR training program would have an enormous possible benefit for little cost.

## Data Availability

Data available upon request.
